# Current Perspectives on Management of Type 2 Diabetes in Youth

**DOI:** 10.3390/children8010037

**Published:** 2021-01-10

**Authors:** Sachi Singhal, Seema Kumar

**Affiliations:** 1 Department of Internal Medicine, Crozer-Chester Medical Center, Upland, PA 19015, USA; Sachi.Singhal@crozer.org; 2 Division of Pediatric Endocrinology, Mayo Clinic, Rochester, MN 55902, USA

**Keywords:** type 2 diabetes mellitus, youth, adolescents

## Abstract

The prevalence of type 2 diabetes mellitus (T2DM) in children and adolescents is on the rise, and the increase in prevalence of this disorder parallels the modern epidemic of childhood obesity worldwide. T2DM affects primarily post-pubertal adolescents from ethnic/racial minorities and those from socioeconomically disadvantaged backgrounds. Youth with T2DM often have additional cardiovascular risk factors at diagnosis. T2DM in youth is more progressive in comparison to adult onset T2DM and shows lower rates of response to pharmacotherapy and more rapid development of diabetes-related complications. Lifestyle modifications and metformin are recommended as the first-line treatment for youth with T2DM in the absence of significant hyperglycemia. Assessment of pancreatic autoimmunity is recommended in all youth who appear to have T2DM. Pharmacotherapeutic options for youth with T2DM are limited at this time. Liraglutide, a GLP-1 agonist, was recently approved for T2DM in adolescents 10 years of age and older. Several clinical trials are currently underway with youth with T2DM with medications that are approved for T2DM in adults. Bariatric surgery is associated with excellent rates of remission of T2DM in adolescents with severe obesity and should be considered in selected adolescents.

## 1. Introduction

The prevalence of childhood obesity is increasing globally, both in the developing and developed nations [1]. Obesity is a significant risk factor for type 2 diabetes and the increase in obesity has paralleled the increase in prevalence of type 2 diabetes (T2DM) [2]. In the SEARCH for Diabetes in Youth study in the United States, the unadjusted incidence rate of T2DM increased from 9.0 cases per 100,000 in 2002–2003 to 12.5 cases per 100,000 in 2011–2012 [3]. The adjusted relative annual increase of T2DM of 4.8% was highly significant. The SEARCH study also reported that the prevalence of T2DM in youth (defined as <20 years of age) increased by 30.5% (95% CI 17.3–45.1) between 2001 and 2009, after adjustment for case ascertainment [4]. T2DM disproportionately affects racial and ethnic minorities including Native Americans, Non-Hispanic Blacks, Hispanics, Asians, and Pacific Islanders [3,4]. Additionally, T2DM occurs in youth from disadvantaged backgrounds where multiple challenges to healthy lifestyle and adherence to medical recommendations often exist. Increase in the incidence of T2DM amongst children has been reported from other countries as well [5,6,7].

T2DM in youth differs from T2DM in adults with regard to the durability of glycemic control with lower rates of response to initial therapy such as lifestyle modifications and metformin [8,9,10]. Additionally, complications related to youth onset T2DM occur earlier than in adults and are often present at the time of diagnosis of diabetes [11,12,13]. Youth with T2DM also have other cardiovascular risk factors such as hypertension [11] and dyslipidemia [14] that further increase their risk for cardiovascular disease.

T2DM in youth should be diagnosed using the criteria proposed by the American Diabetes Association (ADA) criteria [15]. Fasting plasma glucose (FPG) ≥126 mg/dL or 2-h glucose concentration during an oral glucose tolerance test ≥200 mg/dL or hemoglobin A1C (A1C) ≥6.5% can be used as the criteria. The testing should be confirmed with a repeat test on a different day if the patient is asymptomatic. Random plasma glucose ≥200 mg/dL in a patient with symptoms of hyperglycemia is consider diagnostic of diabetes.

**Figure 1:**
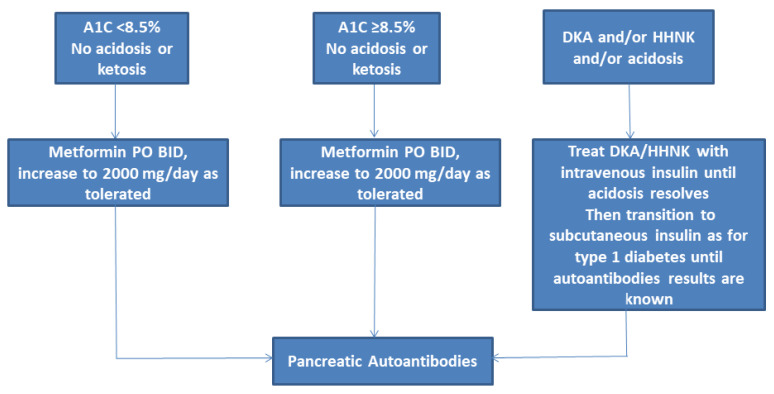
Initial management of new onset diabetes in youth with overweight or obesity. DKA, diabetic ketoacidosis; HHNK, hyperosmolar hyperglycemic nonketotic syndrome. Adapted from American Diabetes, A., 13. Children and Adolescents: Standards of Medical Care in Diabetes-2020. *Diabetes Care* **2020**, *43* (Suppl. 1), S163–S182.

Current recommendations are to obtain pancreatic autoantibodies on all children in whom the diagnosis of T2DM is being considered as antibody-positive youth progress to insulin requirement more rapidly and are at risk for autoimmune disorders [16] (Figure 1). Additionally, due to the high prevalence of obesity in adolescents, the presence of overweight/obesity itself does not rule out type 1 diabetes. T2DM is extremely unlikely in prepubertal children. Testing for monogenic diabetes should also be considered in selected youth [15].

T2DM in youth should be managed by a multidisciplinary team consisting of a physician/other medical provider with expertise in diabetes, dietitian, nurse educator, mental health professional, and exercise specialists, if possible. The overarching goals of management of youth with T2DM are to achieve and subsequently maintain glycemic control, identify and manage associated comorbid conditions, and ultimately prevent microvascular and macrovascular complications of diabetes [15,17].

Glycemic Targets: A reasonable goal for most youth with T2DM is A1C less than 7% or FPG < 130 mg/dL [15]. However, more stringent goals such as less than 6.5% may be appropriate for selected individuals if the goal can be met without significant hypoglycemia or other adverse effects and in those with short duration of diabetes or patients who receive therapy with lifestyle or metformin only with significant weight improvement [15]. The lower target of <6.5% in selected instances is supported by the evidence that hypoglycemia in patients with T2DM receiving insulin is rare [18] and A1C > 6.3% after 3 months of metformin or any increasing A1C even in the non-diabetes range was associated with increased risk for loss of glycemic control in the TODAY study [10]. The A1C targets should also be individualized based on baseline A1C at the time of diagnosis. Higher A1C targets in the short term might be appropriate for those that present with significantly elevated A1C at baseline. Self-monitoring of blood glucose needs to be individualized depending on the type of intervention for T2DM.

## 2. Lifestyle Modifications

Lifestyle modifications are recommended in all youth with T2DM at diagnosis [15,17] (Figure 1). The interventions should be provided with culturally and developmentally appropriate comprehensive lifestyle programs that integrate diabetes management with the ultimate goal of 7–10% reduction in body weight in those that have completed linear growth [15]. Weight loss of the magnitude of 0.5 kg/m^2^ decrease in body mass index (BMI) [19] or a 0.25 to 0.5 decrease in BMI Z-score has been in children has been associated with improvements in insulin sensitivity [20].

Nutritional recommendations should focus on healthy eating patterns with an emphasis on the consumption of nutrient dense high-quality foods and decreased consumption of calorie-dense, nutrient poor foods [15,21]. Important strategies to facilitate these changes in eating habits include reduction in portion size, decreasing frequency of eating out, and replacing or eliminating high-calorie beverages such as sugar-containing drinks and juices with water or calorie-free beverages [22]. There is a lack of data on the effect of any specific type of dietary regimen on glycemic control in adolescents with T2DM. Data on the short-term or long-term efficacy of very low-calorie diets in this population is also minimal. In one study of 20 adolescent patients with T2DM, treatment with very low-calorie diets was associated with significant decrease in hemoglobin A1c from 8.8 to 7.4% [23]. Pharmacological agents were discontinued in all but one of the 20 participants.

The physical activity goals for children and adolescents with T2DM are the same as those for youth in general [24]. Youth with T2DM should be encouraged to engage in moderate to vigorous physical activity for at least 30–60 min at least 5 days per week [25]. Examples of moderate activity include games such as baseball, catch and softball, hiking, brisk walking, and skateboarding. Examples of vigorous activities include games such as sports such as soccer, basketball, and ice or field hockey, jumping rope, and running. Resistance training (also called strength training) is recommended at least three times weekly. Resistance training can include muscle-strengthening activities such as weight lifting, push-ups, pull-ups, climbing ropes, and bone strengthening activities such as skipping, running, and jumping rope.

Both aerobic and resistance training improve insulin action by increasing glucose uptake by muscle [24]. A combination of aerobic and resistance exercise training may be more effective for glucose control than either type of exercise alone [26]. There are no data on the effect of resistance training on glycemic control in adolescents with T2DM but data in overweight and obese adolescents reveal improvement in insulin sensitivity with resistance training [27,28]. Twice per week resistance training in overweight Latino adolescents for a period of 12 weeks resulted in a significant improvement in insulin sensitivity relative to non-exercising adolescents in the control group [27]. The improvement in insulin sensitivity has been shown to be independent of changes in body composition [27,28]. Sedentary behavior should be decreased, and non-academic screen time such as computer, television, and video game should be restricted to less than 2 h daily [25]. Increase in physical activity has been shown to improve insulin sensitivity independent of its effect on body weight [29]. Additionally, exercise leads to improvement in cardiovascular risk factors, reduction in weight, and improvement in well-being [30]. Since many youth with T2DM may be sedentary at the time of diagnosis, assessment of physical activity at baseline and gradual increase to the target may be recommended to avoid musculoskeletal injury and improve adherence.

The TODAY study was a landmark study that enrolled 699 youth with T2DM who were between the ages 10 and 17. They had to have T2DM of less than 2 years duration and body mass index at or above the 85th percentile. All participants had no evidence for pancreatic autoimmunity and were C-peptide positive. After a run in period of 2–6 months during which study subjects received metformin alone and standard diabetes education on healthy eating and exercise and achieved an A1C of less than 8% [31], subjects were randomized to one of three arms, metformin alone, metformin plus insulin sensitizer rosiglitazone, or metformin plus intensive lifestyle intervention. The principal goal of the intensive lifestyle program in the study was to decrease percent overweight defined as BMI minus BMI at the 50th percentile for age and sex, divided by BMI at the 50th percentile times 100 by ≥7% through changes in eating and physical activity habits and to sustain those changes via ongoing treatment contact. The physical activity target for the intensive lifestyle program was 200 min per week of moderate–vigorous intensive activity for most participants and up to 300 min per week for those participants who entered the study already engaging in some regular, physical activity. The lifestyle intervention was delivered during face-to-face counseling sessions for the first 2 years [32]. The primary outcome was treatment failure or loss of glycemic control (defined as A1C ≥8% for at least 6 months or sustained metabolic decompensation requiring initiation of insulin for 3 months or more).

The rates of treatment failure were 51.7% with metformin alone, 46.6% with metformin plus intensive lifestyle intervention, and 38.6% with metformin plus rosiglitazone [33]. Therefore, lifestyle interventions did not confer an additional advantage in terms of glycemic control over metformin alone. Lifestyle intervention also did not confer an additional benefit in terms of weight loss, with 24.3% reaching weight loss target with metformin alone, 31.2% with metformin and intensive lifestyle intervention, and 16.7% with metformin in combination with rosiglitazone [33]. The lack of additional benefit of intensive lifestyle intervention to metformin for both glycemic control and weight loss may be partly related to the low rate of attendance at lifestyle program visits with only half of the participants achieving the preplanned target of attending 75% or more of the visits over a period of 2 years [33]. Multicomponent lifestyle interventions have been shown to have only modest weight loss effect in adolescents with severe obesity [34,35].

## 3. Pharmacological Interventions

### 3.1. Metformin

Current guidelines recommend the initiation of pharmacotherapy for children and adolescents at time of diagnosis of T2DM, along with intensive lifestyle management and diabetic education (Figure 1) [15]. Metformin is the preferred drug in youth that present with A1C < 8.5% and are in no acidosis or ketosis. Additionally, asymptomatic adolescents with A1C ≥ 8.5% may receive a trial of metformin if the provider anticipates adherence to lifestyle modifications. Metformin is a biguanide that decreases hepatic glucose production by inhibiting gluconeogenesis (Table 1) [36,37,38]. The effect on gluconeogenesis is mediated via the inhibition of a specific mitochondrial isoform of glycerophosphate dehydrogenase (mGPD), an enzyme responsible for converting glycerophosphate to dihydroxyacetone phosphate, thereby preventing glycerol from contributing to the gluconeogenic pathway. The inhibition of mGPD leads to an accumulation of cytoplasmic NADH and a reduced conversion of lactate to pyruvate, resulting in decreased lactate contribution to hepatic glucose production. This leads to a release of excess glycerol and lactate into the plasma.

In the TODAY study, almost half of the subjects (48.3%) on metformin alone were able to maintain adequate glycemic control defined as A1C < 8% on metformin alone for up to 6 months [33]. In the half that did not achieve glycemic control on metformin alone, the median time to treatment failure was 11.8 months [33]. Therefore, the likelihood of youth requiring additional pharmacological treatment to meet glycemic controls was much higher than in adults. Metformin was not associated with any improvement in beta cell function with youth with T2DM, unlike in adults [16].

Metformin is associated with modest weight loss. In the TODAY trial, 24.3% of subjects on metformin alone reached the weight loss target (reduction of at least 7 percentage points in % overweight defined as BMI minus BMI at the 50th percentile for age and sex, divided by BMI at the 50th percentile) and 31.2% of those on metformin and intensive lifestyle intervention reached the weight loss target [33].

Metformin should be started at a dose of 500–1000 mg per day and be gradually increased to a recommended therapeutic dose of 1000 mg twice daily by escalating every 1–2 weeks. Metformin is associated with gastrointestinal side effects, and therefore, some patients may require slower dose escalation or may not be able to tolerate the maximum dose. Extended-release metformin preparations may have less frequent gastrointestinal side effects and are now currently available in tablet as well as in suspension form. These extended-release preparations are also beneficial for those who have difficulty complying with twice daily medications. Contraindications for metformin include hepatitis, renal insufficiency, cirrhosis, cardiopulmonary insufficiency, or alcoholism, as metformin can lead to lactic acidosis in the settings. The absorption of vitamin B12 and folic acid can be impaired in patients taking metformin. Therefore, adolescents taking metformin should be advised to take a daily multivitamin. All females with T2DM should be counseled regarding the need for birth control, as metformin can lead to the normalization of irregular menses and ovulatory cycles in females with polycystic ovary syndrome. Metformin should be discontinued 24 h before elective surgery and resumed 48 h after the procedure provided there are no complications.

### 3.2. Liraglutide

Glucagon-like peptide (GLP-1) is produced by the L cells of the small intestine and is secreted following the ingestion of nutrients. GLP-1 stimulates glucose-dependent insulin release from the pancreatic beta cells and inhibits post-meal glucagon release (Table 1) [39]. GLP-1 also has been shown to slow gastric emptying. Liraglutide is a GLP-1 analog that was approved by the Food and Drug Administration (FDA) and European Medicines Agency (EMA) in 2019 for children ≥10 years and adolescents with T2DM. This approval was based on data from the recent ELLIPSE trial [40]. Similar to actions of endogenous GLP-1, liraglutide potentiates the post-prandial release of insulin, inhibits glucagon release, and increases satiety. GLP-1 analogs have been associated with modest weight loss in adolescents with obesity without T2DM [41,42]. In the ELLIPSE trial, 135 adolescents with T2DM between the ages of 10 and 16 years (mean age 14.6 years) who had glycated hemoglobin levels between 7 and 11% if they were being treated with diet or exercise alone or between 6.5 and 11% if they were being treated with metformin with or without insulin were randomized to subcutaneous liraglutide (up to 1.8 mg daily) or placebo for 26 weeks plus metformin with or without basal insulin in combination with diet and exercise regimen [40]. At 26 weeks, the mean glycated hemoglobin decreased by 0.64 percentage points with liraglutide and rose by 0.42 percentage points with placebo (estimated treatment difference: −1.06 percentage points, *p* < 0.001). By 52 weeks, estimated treatment difference had increased to −1.3 percentage points (decrease of −0.5 in liraglutide group and increase of 0.8 in the placebo group). Glycated hemoglobin levels of less than 7% were achieved in 63.7% of the patients in the liraglutide group as compared with 36.5% in the placebo group (*p* < 0.001). The fasting plasma glucose was lower by 1.08 mmol/L at 26 weeks and by −1.03 mmol/L at 52 weeks in the liraglutide group and higher by 0.8 mmol/L at 26 weeks and by 0.78 mmol/L at 52 weeks in the placebo group. Both treatment arms had decreases in BMI, and the change in BMI z-score was not significantly different between the two groups (decrease by 0.25 at 6 months and by 0.34 at 52 weeks in the liraglutide group and decrease of 0.21 at 26 weeks and 0.16 at 52 weeks in the placebo group). The rate of adverse events per 1 patient-year of exposure was higher with liraglutide than with placebo due to a higher incidence of gastrointestinal adverse events, especially during the initial 8 weeks. Nausea was the most frequently reported adverse event. Hypoglycemic episodes and incidence of hypoglycemia were higher with liraglutide.

**Figure 2:**
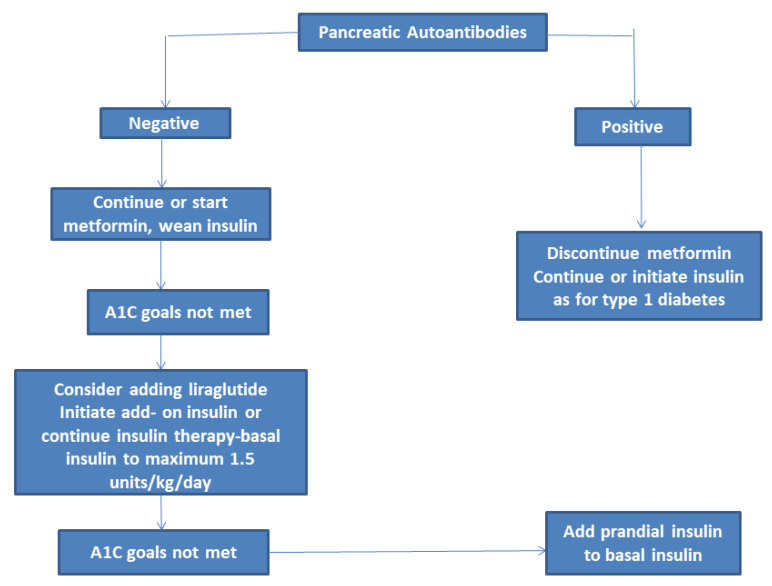
Goal-directed management of type 2 diabetes mellitus (T2DM) in youth. Adapted from American Diabetes, A., 13. Children and Adolescents: Standards of Medical Care in Diabetes-2020. *Diabetes Care* **2020**, *43* (Suppl. 1), S163–S182.

Current guidelines recommend considering liraglutide if glycemic targets are not met with metformin with or without basal insulin in children 10 years of age and older if they have no past medical history or family history of medullary carcinoma, multiple endocrine neoplasia type 2, or pancreatitis [25] (Figure 2). Liraglutide is started at a dose of 0.6 mg subcutaneously once daily. The dose is increased by 0.6 mg increments every 1–2 weeks or longer until fasting glucose targets are achieved to a maximum of 1.8 mg daily. Liraglutide has been noted to be associated with lower rates of adverse cardiovascular outcomes and the development and progression of diabetic kidney disease in adults with type 2 diabetes [43,44]. Currently, clinical trials are underway with other GLP-1 agonists for youth aged 10–17 years, dulaglutide (NCT02963766), and once weekly exenatide (NCT01554618).

### 3.3. Thiazoledinediones

Thiazolidinediones (TZDs) are agonists of peroxisome proliferator-activated receptors (PPARs), mostly PPAR-gamma that increase insulin sensitivity by their effect on adipose tissue and muscle to increase glucose utilization (Table 1) [45,46]. These drugs also decrease hepatic glucose production [46].

In the TODAY study, the metformin plus rosiglitazone arm had the lowest failure rate (loss of glycemic control defined as A1C ≥8% for at least 6 months or sustained metabolic decompensation leading to insulin use for three months or more) of 38.6% in comparison to metformin alone (51.7%) and metformin plus lifestyle modifications (46.6%) [33]. However, TZDs are not recommended for treatment of T2DM in youth, as the use of this class of medications was subsequently restricted by the FDA due to cardiovascular concerns with rosiglitazone [47], bladder cancer concerns with pioglitazone [48], and increased fracture risk concerns with both [49]. Other adverse effects noted include weight gain, fluid retention, and anemia.

### 3.4. Sulfonylureas

Sulfonylureas stimulate the release of insulin from the pancreatic beta cells by interacting with the sulfonylurea receptor of the cells, thereby leading to inhibition of the adenosine triphosphate (ATP)-sensitive potassium channel (K-ATP channel) in the cells (Table 1) [50]. In a randomized trial (glimepiride versus metformin) in 285 youth with T2DM (mean age 13.8 ± 2.3 years and baseline A1C 8.5 ± 1.58%), participants receiving glimepiride (1–8 mg once daily) had a 0.54% reduction in A1C, and 42.4% of subjects in that group achieved A1C < 7% at 24 weeks [51]. The reduction in A1C in the glimepiride was similar to the group receiving metformin (500–1000 mg twice daily). However, weight gain was noted in the glimepiride group (BMI change 0.26 kg/m^2^ for glimepiride versus −0.33 kg/m^2^ for metformin, *p* = 0.003). There were no differences in the rate of hypoglycemia between the two groups.

### 3.5. Dipeptidyl Peptidase-4 (DPP-4) Inhibitors

Dipeptidyl peptidase-4 (DPP-4) is an enzyme that deactivates several bioactive peptides, including glucose-dependent insulinotropic polypeptide (GIP) and GLP-1. DPP-4 inhibitors act by prolonging the action of endogenous GLP-1 and GIP, translating in a glucose-appropriate increase in insulin secretion and suppression of glucagon release (Table 1) [52]. In a dose-finding study of linagliptin in 39 adolescents with T2DM, linagliptin use was associated with a dose-dependent decrease in A1C and fasting glucose [53]. The A1C reduction (compared to placebo) at 12 weeks from baseline was 0.48% and 0.63% with linagliptin 1 mg and 5 mg respectively. Reduction in mean fasting plasma glucose was 5.6 and 34.2 mg/dL in the 1 mg and 5 mg linagliptin groups, respectively. A phase 3 study (DINAMO)TM comparing linaglipin 5 mg with SGLT2 inhibitor empagliflozin and placebo is ongoing (NCT 03429543). Side effects reported in adults with use of DPP-4 inhibitors include upper respiratory infection, headache, and hypersensitivity reactions [54]. No drug-related adverse effects were reported in the pediatric study [53]. Several trials in youth with T2DM with sitagliptin (NCT01760447, NCT01472367, and NCT01485614) have been recently completed, and another trial with alogliptin is ongoing (NCT02856113).

### 3.6. Sodium-Glucose Co-Transporter 2 (SGLT2) Inhibitors

Sodium-glucose co-transporter 2 (SGLT2) inhibitors mediate their effect by inhibiting the SGLT-2 present in the proximal collecting tubule of the nephron. These medications reduce the reabsorption of glucose and thereby increase urinary glucose excretion (Table 1). Glucosuria leads to an improvement in blood glucose levels and A1C and reduction in weight [55]. In a single dose, open-label, randomized parallel group study in youth with T2DM aged 10–17 years with empagliflozin 5, 10, and 25 mg, exposure response relationships were noted to be similar between youth and adults after adjusting for significant covariates [56]. The drug was well tolerated with no adverse events, and one mild “dehydration” physician-reported event was reported in the small cohort [56]. In adults, reported adverse effects of SGLT2 inhibitors include vulvovaginal candidiasis, urinary tract infections, hypotension, and amputations [57]. SGLT2 inhibitors appear to increase the risk of diabetic ketoacidosis [58]. Many SGLT2 inhibitors have been shown to benefit cardiovascular and renal outcomes in adults with T2DM [59,60]. Currently, two clinical trials are studying other SGLT2 inhibitors in youth/young adults: ages 10–24; dapaglifozin (NCT02725593), and ages 10–17 years, canagliflozin (NCT03170518). Two ongoing clinical trials are examining the combination of DPP-4 inhibitors and SGLT2 inhibitors (linagliptin plus empagliflozin NCT03429543 and dapagliflozin plus saxagliptin NCT03199053) in youth with T2DM.

### 3.7. Insulin

The ADA guidelines recommend the initiation of insulin in youth that present with ketosis/ketonuria/ketoacidosis/hyperosmolar hyperglycemic non-ketotic syndrome or those with A1C ≥ 8.5% [15,17,25] (Figure 2). In these patients, subcutaneous or intravenous insulin should be initiated initially for the correction of hyperglycemia and metabolic abnormalities. Subcutaneous insulin should be continued and metformin can be started once the acidosis has resolved.

In youth that have A1C ≥ 8.5% without acidosis or ketosis, basal insulin is recommended at a starting dose of 0.25–0.5 units/kg/day, and the dose can be adjusted based on blood glucose values (Table 1). Long acting once-daily insulin preparations such as insulin glargine, detemir, or degludec can be started at bedtime. Metformin can also be started if acidosis or ketosis are not present.

The guidelines prior to the FDA approval of liraglutide for adolescents 10 and older with T2DM recommended that insulin should be considered if glycemic targets are not met within 4 months on metformin monotherapy [15,17]. However, liraglutide can now considered as an option in this scenario before starting insulin [25].

Multiple daily injections with prandial short-acting insulin should be recommended in youth on high doses of basal insulin (up to 1.5 units/kg/day). Pancreatic autoantibodies are helpful in the management of diabetes in youth. If autoantibodies are positive, insulin therapy should be continued or initiated, and metformin should be discontinued (Figure 2). If pancreatic autoantibodies are negative, metformin should be continued or started, and an attempt should be made to wean insulin guided by self-monitoring of blood glucose (SMBG) values. Most (90%) of the youth in the TODAY study with recent onset T2DM were able to be weaned off insulin and treated with metformin alone [31,61]. There is need for data on use of continuous glucose monitoring (CGM) in youth with T2DM that are on intensive insulin therapy [62]. In adults with T2DM, the use of CGM was associated with an improvement in A1C at 24 weeks compared to the control group [63]. Adverse effects related to insulin use include weight gain and hypoglycemia. Hypoglycemia is rare in youth with T2DM due to insulin resistance [18]. Insulin regimens should be carefully tailored to lifestyle and individual needs.

In conclusion, the initiation of metformin in conjunction with lifestyle interventions is recommended in all children with T2DM in the absence of acidosis/ketosis. Insulin therapy is recommended as the first line in those that present initially in diabetic ketoacidosis or hyperosmolar hyperglycemic nonketotic syndrome. Metformin can be added in those that were started on insulin initially after the resolution of acidosis/ketosis. Liraglutide and/or insulin should be considered as add-on second-line medications in youth that are not able to achieve optimal glycemic control on metformin alone. Ongoing trials are studying the effect of other GLP-1 analogs, SGLT2 inhibitors, and DPP-4 inhibitors in youth with T2DM. The results from these trials would expand the availability of pharmacological options for youth with T2DM in the near future.

**Table 1:** Pharmacologic agents (Food and Drug Administration (FDA)-approved and off-label treatments for type 2 diabetes in children).

Drug Name	Mechanism of Action		FDA Indication	Dose	Side Effects
Metformin	Increases insulin mediated glucose uptake in peripheral tissues and decreases hepatic glucose production		T2DM ≥ 10 years	Start at 500–1000 mg per day, Increase to 2000 mg/day in 1–2 divided doses	Diarrhea, nausea, epigastric discomfort, vomiting, flatulence, B12 deficiency, lactic acidosis
Liraglutide	Increases glucose-dependent insulin release from the pancreatic beta cells and inhibits post-meal glucagon release, also slows gastric emptying		T2DM ≥ 10 years of age	Start at 0.6 mg subcutaneously once daily. Increase by 0.6 mg increments every 1–2 weeks or longer to a maximum of 1.8 mg daily	Nausea, vomiting, abdominal pain, diarrhea, potential hypoglycemia
Rosiglitazone	Agonist of peroxisome proliferator-activated receptors (PPARs), mostly PPAR gamma that increases insulin sensitivity by its effect on adipose tissue and muscle to increase glucose utilization; also decreases hepatic glucose production		Not approved in adolescents	Start at 2 mg twice daily, then increase to 4 mg twice daily after 8 weeks	Weight gain, fluid retention, decreased bone density, heart failure, anemia
Glimepiride	Stimulation of insulin secretion via inhibition of the adenosine triphosphate (ATP) sensitive potassium channel (K-ATP channel) in the pancreatic beta cells	Not approved in adolescents		Start at 1 mg daily, can increase in 1–2 mg increments to maximum of 8 mg daily	Weight gain, hypoglycemia
Linagliptin	Prolonging the action of endogenous GLP-1 and GIP, translating in a glucose-appropriate increase in insulin secretion and suppression of glucagon release	Not approved in adolescents		1 and 5 mg daily were used in one study [53] (5 mg was more effective in decreasing fasting blood glucose)	Upper respiratory infection, headache, and hypersensitivity reactions
Empagliflozin	Decreases reabsorption of glucose and thereby increases urinary glucose excretion	Not approved in adolescents		Dose in adults: start at 10 mg daily, can increase to 25 mg once daily after 4–12 weeks	Vulvovaginal candidiasis, urinary tract infections, hypotension, risk of diabetic ketoacidosis
Insulin: Basal (such as Glargine, Detemir, Degludec) and short acting (Aspart, Lispro)	Facilitates cellular uptake of glucose into myofibers and adipocytes and stimulates GLUT4 translocation	Approved for adolescents with T2DM		Basal insulin start at 0.25–0.5 units/kg/day and increase based on blood glucose. Dose of short acting is determined by blood glucose values	Hypoglycemia, weight gain, hypertrophy/lipoatrophy at injection site

## 4. Metabolic Surgery

Metabolic surgery should be considered for those adolescents with T2DM that have severe obesity (BMI ≥ 35 kg/m^2^) and have uncontrolled glycemic control and/or serious comorbidities despite lifestyle and pharmacological interventions [15,17,22,25].

Bariatric surgery is highly effective in leading to remission or improvement in T2DM in youth with severe obesity [64,65,66,67]. Improvement in insulin sensitivity and improvement in beta cell function were noted following Roux-en-Y gastric bypass surgery in severely obese adolescents and young adults without diabetes [68]. In a study of 29 adolescents with severe obesity and T2DM enrolled in the Teen Longitudinal Assessment of Bariatric Surgery (Teen-LABS) study who underwent Roux-en-Y gastric bypass or sleeve gastrectomy, remission of diabetes was noted in 95% of the patients at 3 years after the procedure [64]. Remission of pre-diabetes was noted in 76% of the cohort. Amongst a subset that had undergone Roux-en-Y gastric bypass surgery, an 84% reduction in the prevalence of T2DM was noted at a mean follow-up of 8 years (SD 1.6, range 5.4–12.5) [65]. A series of 108 patients between the ages of 5 through 21 years reported 91% remission of diabetes and 100% remission of prediabetes following laparoscopic sleeve gastrectomy [69]. The rates of remission of T2DM in adolescents were noted to be higher in adolescents than in adults (86% vs. 53%, risk ratio 1.27; 95% CI 1.03 to 1.57) despite no differences in percent weight change [70]. In another study, relapse of diabetes 5 years after laparoscopic sleeve gastrectomy was seen in 13% of diabetic adults, but none of the adolescents relapsed [67]. In a secondary analysis of data from severely obese adolescents that underwent surgical intervention (Teen-LABS, 30 subjects, mean age 16.9 [13] years) and medical interventions (TODAY consortia, 63 subjects, mean age 15.3 [13] years) over a period of two years, mean A1C decreased from 6.8% (95% CI, 6.4−7.3%) to 5.5% (95% CI, 4.7% to 6.3%) in Teen-LABS and went up from 6.4% (95% CI, 6.1−6.7%) to 7.8% (95% CO, 7.2−8.3%) in TODAY [71].

Bariatric surgery leads to significant weight loss and improvement in several cardiovascular risk factors in adolescents with severe obesity [64,65,69,70,72]. In the Teen-LABS study with 242 adolescents (*n* = 161 for Roux-en-Y gastric bypass and *n* = 67 for sleeve gastrectomy), at 3 years after surgery, 27% weight loss (28% among those who underwent gastric bypass and 26% among those who underwent sleeve gastrectomy) was documented [64]. BMI reduction of 29.2% was noted at 5 years in 58 individuals undergoing RYGB [65]. Remission of elevated blood pressure was seen in 74% (95% CI, 64 to 84), and remission of dyslipidemia in 66% (95% CI, 57 to 74) at 3 years following surgery [64]. Significant improvements in blood pressure and lipids were also observed at mean follow up of 8 years in adolescents who underwent Roux-en-Y gastric bypass surgery [65].

Other eligibility criteria for bariatric surgery include the following: (a) History of previous sustained efforts to lose weight through changes in diet and physical activity; (b) Commitment from the patient and family to adhere to recommended pre- and postoperative treatments, including vitamin and mineral supplementation; and (c) Patient and family understanding of the risks and benefits of bariatric surgery [73,74].

The most recent expert guidelines do not recommend withholding surgery in those that have not completed puberty or physical maturity as determined by Tanner stage or skeletal maturation [73,74]. Contraindications for bariatric surgery include the following:

(1) Medically correctable cause of obesity;
(2) An ongoing substance abuse problem (within the preceding year);
(3) A medical, psychiatric, psychosocial, or cognitive condition that prevents adherence to postoperative dietary and medication regimens or impairs decisional capacity;
(4) Current or planned pregnancy within 12 to 18 months of the procedure; and
(5) Inability on the part of the patient or parent to comprehend the risks and benefits of the surgical procedure

Adolescents undergoing bariatric surgery need long-term monitoring, as long-term safety and efficacy data in this particular age group are limited [73,74]. Hypoferritinemia is the most common nutritional deficiency noted in adolescents following bariatric surgery in adolescents [64,65]. Other deficiencies include that of vitamin D, vitamin B12, folate, and vitamin A [64,65].

## 5. Assessment of Comorbid Conditions

### 5.1. Dyslipidemia

Youth with T2DM often have dyslipidemia with elevated triglycerides, low high-density lipoprotein cholesterol (HDL-C) and occasional high low-density lipoprotein cholesterol (LDL-C). In the TODAY study, 4.5% of participants were either receiving lipid-lowering therapy or had LDL-cholesterol ≥ 130 mg/dL at baseline, and this number increased over a period of 36 months to 10.7% [14]. Triglycerides ≥ 150 mg/dL were seen in 21% at baseline and increased to 23% over 36 months. The ADA recommendations suggest that lipid testing be performed at diagnosis of diabetes (but after glycemic control is well established) and annually thereafter [25]. Target lipid levels are LDL-C < 100 mg/dL, HDL-C > 35 mg/dL, and triglycerides < 150 mg/dL.

### 5.2. Hypertension

Primary hypertension is prevalent in youth with T2DM. Hypertension was present in 12% of youth in the TODAY study at the time of enrollment and in 34% after an average follow up of 4 years [11]. Blood pressure should be checked at each health care visit, and the goal is BP < 120/80 mmHg or <90th percentile in children younger than 13 years [25].

### 5.3. Nonalcoholic Fatty Liver Disease

Nonalcoholic fatty liver disease is common in children with T2DM and obesity [75]. Abdominal examination for hepatomegaly and measurement of serum alanine transferase (ALT) is recommended in youth with T2DM [25].

## 6. Assessment of Complications

Albuminuria, a sign of early diabetic nephropathy, is common among adolescents with T2DM at diagnosis, and the prevalence increases rapidly [11,75,76]. In the TODAY study, prevalence at baseline was 6% and increased to 18% by 5 years [77]. The urine albumin to creatinine ratio should be obtained at the time of diagnosis and annually. Physical examination should include detailed neurologic examination for neuropathy and should be performed at diagnosis and annually thereafter.

Patients should be screened for obstructive sleep apnea, depression/anxiety, eating disorders, and impairment of cognition at the time of diagnosis.

## 7. Conclusions

The prevalence of T2DM in children and adolescents is increasing due to the epidemic of childhood obesity. T2DM disproportionately affects children from ethnic minorities. Youth with T2DM often have additional cardiovascular risk factors. T2DM in youth is progressive and shows lower rates of response to oral pharmacotherapy than in adults. Lifestyle modifications and metformin are the recommended first-line treatment in children with T2DM in the absence of significant hyperglycemia. Pharmacotherapeutic options for youth with T2DM are limited at this time. However, several clinical trials with agents that are approved for adults are ongoing. Bariatric surgery is associated with excellent rates of remission of T2DM in adolescents and should be considered in selected adolescents with severe obesity.
